# 2-Phenyl-4,5-di-2-pyridyl-1*H*-imidazole

**DOI:** 10.1107/S1600536809053215

**Published:** 2009-12-16

**Authors:** Marika Felsmann, Diana Schindler, Edwin Weber

**Affiliations:** a Institut für Organische Chemie, TU Bergakademie Freiberg, Leipziger Strasse 29, D-09596 Freiberg/Sachsen, Germany

## Abstract

In the title compound, C_19_H_14_N_4_, which was crystallized from dimethyl sulfoxide, the arene and heterocyclic rings of the lophine analogue framework differ only slightly from coplanarity (dihedral angles range from 8.8 to 20.2°), and intramolecular N—H⋯N and  C—H⋯N interactions help to establish the conformation. The crystal packing features a number of weak C—H⋯N, N—H⋯N hydrogen-bond type contacts, and C—H⋯π interactions, leading to the formation of a herringbone structure.

## Related literature

For the solid-state structures of 2,4,5-triphenyl­imidazoles, see: Kaftory *et al.* (1998 ▶); Benisvy *et al.* (2003 ▶); Martinez *et al.* (2004 ▶); Seethalakshmi *et al.* (2006 ▶); Thiruvalluvar *et al.* (2007 ▶). For the synthesis of the title compound, see: Nakashima *et al.* (1998 ▶); Slater *et al.* (2006 ▶). For weak hydrogen-bond type contacts, see: Desiraju & Steiner (1999 ▶).
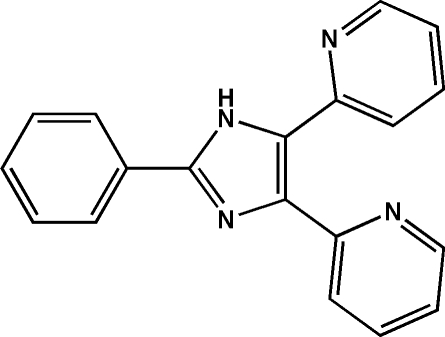


## Experimental

### 

#### Crystal data

C_19_H_14_N_4_
*M* *_r_* = 298.34
Monoclinic, 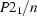

*a* = 8.7394 (3) Å
*b* = 15.3333 (5) Å
*c* = 11.2980 (4) Å
β = 106.835 (2)°
*V* = 1449.09 (9) Å^3^
*Z* = 4
Mo *K*α radiation
μ = 0.08 mm^−1^
*T* = 153 K
0.32 × 0.20 × 0.08 mm

#### Data collection

Bruker SMART CCD area-detector diffractometer
Absorption correction: multi-scan (*SADABS*; Sheldrick, 2004 ▶) *T*_min_ = 0.974, *T*_max_ = 0.993
14338 measured reflections
2562 independent reflections
2130 reflections with *I* > 2σ(*I*)
*R*_int_ = 0.029

#### Refinement

*R*[*F* ^2^ > 2σ(*F* ^2^)] = 0.033
*wR*(*F* ^2^) = 0.084
*S* = 1.04
2562 reflections
212 parameters
H atoms treated by a mixture of independent and constrained refinement
Δρ_max_ = 0.19 e Å^−3^
Δρ_min_ = −0.19 e Å^−3^

### 

Data collection: *APEX2* (Bruker, 2004 ▶); cell refinement: *SAINT* (Bruker, 2004 ▶); data reduction: *SAINT*; program(s) used to solve structure: *SHELXS97* (Sheldrick, 2008 ▶); program(s) used to refine structure: *SHELXL97* (Sheldrick, 2008 ▶); molecular graphics: *ORTEP-3* (Farrugia, 1997 ▶); software used to prepare material for publication: *SHELXTL* (Sheldrick, 2008 ▶).

## Supplementary Material

Crystal structure: contains datablocks global, I. DOI: 10.1107/S1600536809053215/im2166sup1.cif

Structure factors: contains datablocks I. DOI: 10.1107/S1600536809053215/im2166Isup2.hkl

Additional supplementary materials:  crystallographic information; 3D view; checkCIF report

## Figures and Tables

**Table 1:** Hydrogen-bond geometry (Å, °)

*D*—H⋯*A*	*D*—H	H⋯*A*	*D*⋯*A*	*D*—H⋯*A*
N1—H1⋯N3	0.89 (2)	2.37 (2)	2.669 (2)	100 (1)
N1—H1⋯N4^i^	0.89 (2)	2.73 (2)	3.432 (2)	137 (1)
C5—H5⋯N4	0.95	2.31	3.068 (2)	137
C13—H13⋯N3^ii^	0.95	2.72	3.505 (2)	141
C15—H15⋯N4^i^	0.95	2.59	3.442 (2)	149
C7—H7⋯C*g*4^iii^	0.95	2.79	3.708 (1)	163
C11—H11⋯C*g*2^iv^	0.95	2.93	3.772 (1)	148
C16—H16⋯C*g*1^i^	0.95	2.88	3.671 (1)	142

Symmetry codes: (i) 
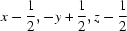
; (ii) 
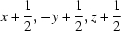
; (iii) 
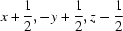
; (iv) 
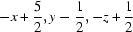
. *Cg*1, *Cg*2 and *Cg*4 are the centroids of the N1/C1,N2,C2,C3, N2/C4–C8 and N4/C9–C13 rings, respectively.

**Fig. 1.**
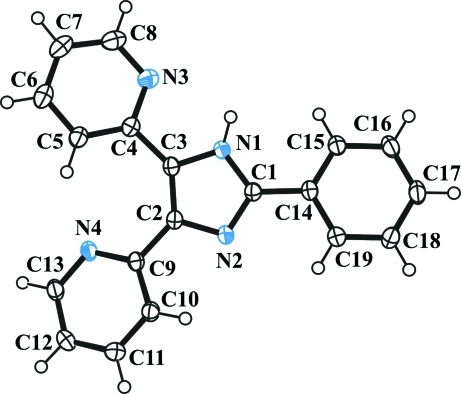
A view of the molecular structure of the title compound, showing 50% probability displacement ellipsoids for the non-hydrogen atoms.

**Fig. 2.**
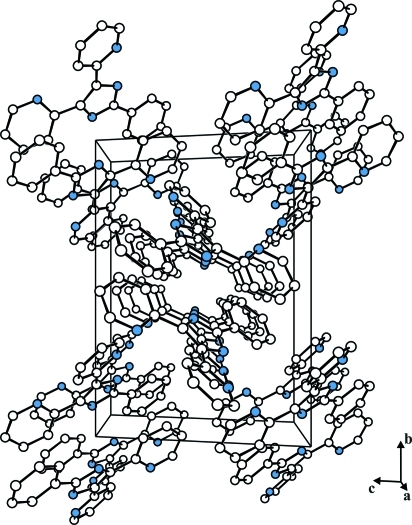
A view of the crystal packing of the title compound, viewed along the a axis. Hydrogen atoms have been omitted for clarity.

## supplementary crystallographic information

### Comment

In the title compound (Fig.1), the imidazole and the phenyl ring are nearly coplanar with a dihedral angle of 8.87 (9)° while the two pyridine rings are tilted showing dihedral angels of 11.14 (8) and 20.17 (8)° with respect to the imidazole unit. As compared with related imidazoles having phenyl substituents in 4- and 5-position (Kaftory *et al.*, 1998; Benisvy *et al.*, 2003; Martinez *et al.*, 2004; Seethalakshmi *et al.*, 2006 and Thiruvalluvar *et al.*, 2007), the dihedral angels involving pyridine rings and imidazole of the present compound are decreased in value of about 20°. This is presumably attributed to the CH for N exchange being connected with the phenyl for pyridyl substitution, which lowers torsional strain between the two planes defining the dihedral angels.

As a consequence of the almost planar overall molecular conformation, weak intramolecular hydrogen bonding contacts (Desiraju & Steiner, 1999) involving the donator and acceptor atoms N1 and N3 as well as C5 and N4, respectively, are rather probable to exist (Table 1).

Due to steric shielding of the nitrogen atoms, strong hydrogen bonds are also absent in the crystal packing. Thus, the nitrogen atoms N3 and N4 of the pyridine rings are acting as hydrogen acceptors and the imidazole nitrogen N1 as a hydrogen donator only in the formation of weak intermolecular C—H···N and N—H···N type contacts (Desiraju & Steiner, 1999) (Table 1). Furthermore the crystal packing is stabilized by weak C—H···π interactions (Table 1) including the three heterocyclic rings, and two *offset-face-to-face* arrangements of the phenyl ring *Cg*2 and the pyridin units *Cg*3 and *Cg*4, [*Cg*2···*Cg*3^v^ = 3.6866 Å and *Cg*2···*Cg*4^vi^ = 3.9773 Å [symmetry operation: (v) 1 + *x*, *y*, *z*; (vi) 2 - *x*, 1 - *y*, -*z*]. They all give rise to the formation of a packing structure being reminiscent of a herring-bone pattern.

### Experimental

The title compound was synthesized from benzaldehyde, 1,2-dipyridin-2-yl-ethane-1,2-dione and ammonium acetate following the literature procedure (K. Nakashima *et al.*, 1998). Yellow prisms suitable for X-ray diffraction were obtained by evaporation of a the solvent from a solution of the title compound in DMSO.

### Refinement

H atoms, except for H1, were positioned geometrically and allowed to ride on their parent atoms, with C–H = 0.95 Å, and *U*_iso_= 1.2–1.5 *U*_eq_ (parent atom).

### Crystal data

**Table:**

C_19_H_14_N_4_	*F*(000) = 624
*M**_r_* = 298.34	*D*_x_ = 1.368 Mg m^−^^3^
Monoclinic, *P*2_1_/*n*	Melting point: 462 K
Hall symbol: -P 2yn	Mo *K*α radiation, λ = 0.71073 Å
*a* = 8.7394 (3) Å	Cell parameters from 4480 reflections
*b* = 15.3333 (5) Å	θ = 2.3–28.9°
*c* = 11.2980 (4) Å	µ = 0.08 mm^−^^1^
β = 106.835 (2)°	*T* = 153 K
*V* = 1449.09 (9) Å^3^	Plate, colourless
*Z* = 4	0.32 × 0.20 × 0.08 mm

### Data collection

**Table:**

Bruker SMART CCD area-detector diffractometer	2562 independent reflections
Radiation source: fine-focus sealed tube	2130 reflections with *I* > 2σ(*I*)
graphite	*R*_int_ = 0.029
phi and ω scans	θ_max_ = 25.0°, θ_min_ = 2.3°
Absorption correction: multi-scan (*SADABS*; Sheldrick, 2004)	*h* = −10→10
*T*_min_ = 0.974, *T*_max_ = 0.993	*k* = −17→18
14338 measured reflections	*l* = −12→13

### Refinement

**Table:**

Refinement on *F*^2^	Primary atom site location: structure-invariant direct methods
Least-squares matrix: full	Secondary atom site location: difference Fourier map
*R*[*F*^2^ > 2σ(*F*^2^)] = 0.033	Hydrogen site location: inferred from neighbouring sites
*wR*(*F*^2^) = 0.084	H atoms treated by a mixture of independent and constrained refinement
*S* = 1.04	*w* = 1/[σ^2^(*F*_o_^2^) + (0.0408*P*)^2^ + 0.3271*P*] where *P* = (*F*_o_^2^ + 2*F*_c_^2^)/3
2562 reflections	(Δ/σ)_max_ < 0.001
212 parameters	Δρ_max_ = 0.19 e Å^−^^3^
0 restraints	Δρ_min_ = −0.18 e Å^−^^3^

### Special details

**Table:**

Geometry. All e.s.d.'s (except the e.s.d. in the dihedral angle between two l.s. planes) are estimated using the full covariance matrix. The cell e.s.d.'s are taken into account individually in the estimation of e.s.d.'s in distances, angles and torsion angles; correlations between e.s.d.'s in cell parameters are only used when they are defined by crystal symmetry. An approximate (isotropic) treatment of cell e.s.d.'s is used for estimating e.s.d.'s involving l.s. planes.
Refinement. Refinement of *F*^2^ against ALL reflections. The weighted *R*-factor *wR* and goodness of fit *S* are based on *F*^2^, conventional *R*-factors *R* are based on *F*, with *F* set to zero for negative *F*^2^. The threshold expression of *F*^2^ > σ(*F*^2^) is used only for calculating *R*-factors(gt) *etc*. and is not relevant to the choice of reflections for refinement. *R*-factors based on *F*^2^ are statistically about twice as large as those based on *F*, and *R*- factors based on ALL data will be even larger.

### Fractional atomic coordinates and isotropic or equivalent isotropic displacement parameters (Å^2^)

**Table:**

	*x*	*y*	*z*	*U*_iso_*/*U*_eq_	Occ. (<1)
N1	0.91201 (12)	0.18912 (7)	−0.10112 (9)	0.0237 (3)	
N2	0.86233 (11)	0.09328 (7)	0.02858 (9)	0.0239 (3)	
N3	1.16211 (13)	0.28374 (8)	−0.11585 (10)	0.0331 (3)	
N4	1.24782 (12)	0.12084 (7)	0.25734 (10)	0.0265 (3)	
C1	0.80100 (14)	0.13607 (8)	−0.07612 (11)	0.0226 (3)	
C2	1.02004 (14)	0.11894 (8)	0.07188 (11)	0.0225 (3)	
C3	1.05460 (14)	0.17873 (8)	−0.00916 (11)	0.0224 (3)	
C4	1.19531 (14)	0.22527 (8)	−0.02296 (11)	0.0239 (3)	
C5	1.35107 (14)	0.20897 (9)	0.04894 (12)	0.0292 (3)	
H5	1.3719	0.1672	0.1138	0.035*	
C6	1.47484 (16)	0.25437 (9)	0.02471 (13)	0.0333 (3)	
H6	1.5820	0.2440	0.0727	0.040*	
C7	1.44232 (17)	0.31470 (10)	−0.06921 (13)	0.0352 (3)	
H7	1.5256	0.3470	−0.0871	0.042*	
C8	1.28509 (18)	0.32678 (10)	−0.13653 (14)	0.0386 (4)	
H8	1.2625	0.3683	−0.2018	0.046*	
C9	1.11465 (14)	0.08007 (8)	0.19008 (11)	0.0224 (3)	
C10	1.06033 (15)	0.00341 (9)	0.23010 (11)	0.0264 (3)	
H10	0.9655	−0.0238	0.1810	0.032*	
C11	1.14491 (15)	−0.03268 (9)	0.34130 (12)	0.0292 (3)	
H11	1.1095	−0.0851	0.3698	0.035*	
C12	1.28242 (15)	0.00858 (9)	0.41110 (12)	0.0295 (3)	
H12	1.3430	−0.0145	0.4885	0.035*	
C13	1.32884 (15)	0.08392 (9)	0.36510 (12)	0.0292 (3)	
H13	1.4242	0.1116	0.4125	0.035*	
C14	0.63692 (14)	0.12433 (8)	−0.15534 (11)	0.0233 (3)	
C15	0.56985 (15)	0.17767 (9)	−0.25665 (11)	0.0267 (3)	
H15	0.6320	0.2225	−0.2782	0.032*	
C16	0.41256 (15)	0.16545 (9)	−0.32612 (12)	0.0318 (3)	
H16	0.3672	0.2020	−0.3953	0.038*	
C17	0.32096 (15)	0.10063 (10)	−0.29574 (12)	0.0329 (3)	
H17	0.2126	0.0932	−0.3431	0.040*	
C18	0.38761 (15)	0.04655 (9)	−0.19604 (12)	0.0322 (3)	
H18	0.3252	0.0016	−0.1752	0.039*	
C19	0.54504 (15)	0.05787 (9)	−0.12681 (12)	0.0286 (3)	
H19	0.5908	0.0200	−0.0593	0.034*	
Cg1	0.9300	0.1432	−0.0172	0.010*	0.00
Cg2	1.3185	0.2690	−0.0452	0.010*	0.00
Cg3	0.4788	0.1121	−0.2261	0.010*	0.00
H1	0.9009 (17)	0.2253 (10)	−0.1644 (14)	0.039 (4)*	
Cg4	1.1965	0.0440	0.2992	0.010*	0.00

### Atomic displacement parameters (Å^2^)

**Table:**

	*U*^11^	*U*^22^	*U*^33^	*U*^12^	*U*^13^	*U*^23^
N1	0.0238 (5)	0.0238 (6)	0.0216 (5)	−0.0007 (4)	0.0034 (4)	0.0019 (5)
N2	0.0211 (5)	0.0254 (6)	0.0235 (5)	−0.0007 (4)	0.0036 (4)	−0.0004 (4)
N3	0.0323 (6)	0.0319 (7)	0.0354 (6)	−0.0016 (5)	0.0106 (5)	0.0060 (5)
N4	0.0214 (5)	0.0279 (7)	0.0274 (5)	0.0009 (4)	0.0026 (4)	0.0008 (5)
C1	0.0225 (6)	0.0215 (7)	0.0228 (6)	0.0003 (5)	0.0053 (5)	−0.0014 (5)
C2	0.0202 (6)	0.0224 (7)	0.0240 (6)	0.0007 (5)	0.0052 (5)	−0.0020 (5)
C3	0.0211 (6)	0.0218 (7)	0.0226 (6)	0.0005 (5)	0.0039 (5)	−0.0031 (5)
C4	0.0255 (6)	0.0216 (7)	0.0255 (6)	−0.0005 (5)	0.0089 (5)	−0.0045 (5)
C5	0.0253 (7)	0.0313 (8)	0.0315 (7)	−0.0010 (6)	0.0088 (5)	−0.0011 (6)
C6	0.0247 (7)	0.0388 (9)	0.0376 (8)	−0.0037 (6)	0.0110 (6)	−0.0072 (7)
C7	0.0343 (8)	0.0358 (9)	0.0415 (8)	−0.0103 (6)	0.0203 (6)	−0.0076 (7)
C8	0.0424 (8)	0.0361 (9)	0.0408 (8)	−0.0056 (7)	0.0174 (7)	0.0076 (7)
C9	0.0197 (6)	0.0234 (7)	0.0242 (6)	0.0029 (5)	0.0064 (5)	−0.0022 (5)
C10	0.0235 (6)	0.0260 (7)	0.0283 (7)	−0.0010 (5)	0.0053 (5)	−0.0011 (6)
C11	0.0302 (7)	0.0264 (8)	0.0317 (7)	0.0006 (6)	0.0101 (6)	0.0045 (6)
C12	0.0284 (7)	0.0310 (8)	0.0264 (7)	0.0066 (6)	0.0035 (5)	0.0040 (6)
C13	0.0229 (6)	0.0319 (8)	0.0285 (7)	0.0018 (5)	0.0006 (5)	0.0001 (6)
C14	0.0226 (6)	0.0242 (7)	0.0226 (6)	0.0015 (5)	0.0055 (5)	−0.0041 (5)
C15	0.0253 (7)	0.0270 (8)	0.0264 (7)	−0.0005 (5)	0.0053 (5)	0.0006 (5)
C16	0.0279 (7)	0.0373 (8)	0.0261 (7)	0.0040 (6)	0.0012 (6)	0.0028 (6)
C17	0.0225 (6)	0.0428 (9)	0.0294 (7)	−0.0018 (6)	0.0012 (5)	−0.0031 (6)
C18	0.0274 (7)	0.0359 (8)	0.0324 (7)	−0.0083 (6)	0.0072 (6)	−0.0015 (6)
C19	0.0275 (7)	0.0305 (8)	0.0257 (7)	−0.0014 (6)	0.0045 (5)	0.0015 (6)

### Geometric parameters (Å, °)

**Table:**

N1—Cg1	1.1544 (11)	C7—H7	0.9500
N1—C1	1.3570 (16)	C8—H8	0.9500
N1—C3	1.3813 (15)	C9—C10	1.3913 (19)
N1—H1	0.888 (16)	C10—C11	1.3759 (17)
N2—Cg1	1.1751 (11)	C10—H10	0.9500
N2—C1	1.3228 (15)	C11—C12	1.3846 (18)
N2—C2	1.3796 (15)	C11—H11	0.9500
N3—C8	1.3382 (18)	C12—C13	1.3753 (19)
N3—C4	1.3465 (16)	C12—H12	0.9500
N4—C13	1.3435 (16)	C13—H13	0.9500
N4—C9	1.3456 (15)	C14—C15	1.3907 (17)
C1—Cg1	1.1364 (11)	C14—C19	1.3919 (18)
C1—C14	1.4650 (16)	C14—Cg3	1.3942 (12)
C2—Cg1	1.1454 (11)	C15—C16	1.3841 (17)
C2—C3	1.3893 (18)	C15—H15	0.9500
C2—C9	1.4774 (16)	C16—C17	1.381 (2)
C3—Cg1	1.1972 (12)	C16—H16	0.9500
C3—C4	1.4686 (17)	C17—C18	1.3841 (19)
C4—Cg2	1.3514 (13)	C17—H17	0.9500
C4—C5	1.3909 (17)	C18—C19	1.3825 (17)
C5—C6	1.3788 (19)	C18—H18	0.9500
C5—H5	0.9500	C19—H19	0.9500
C6—C7	1.374 (2)	Cg2—Cg3^i^	3.6866
C6—H6	0.9500	Cg3—Cg4^ii^	3.9773
C7—C8	1.377 (2)		
			
Cg1—N1—C1	53.07 (7)	N4—C9—C10	121.99 (11)
Cg1—N1—C3	55.48 (7)	N4—C9—C2	119.28 (11)
C1—N1—C3	108.54 (10)	C10—C9—C2	118.70 (11)
Cg1—N1—H1	178.2 (10)	C11—C10—C9	119.53 (11)
C1—N1—H1	128.6 (9)	C11—C10—H10	120.2
C3—N1—H1	122.9 (9)	C9—C10—H10	120.2
Cg1—N2—C1	53.73 (7)	C10—C11—C12	118.97 (13)
Cg1—N2—C2	52.54 (7)	C10—C11—H11	120.5
C1—N2—C2	106.27 (10)	C12—C11—H11	120.5
C8—N3—C4	117.50 (11)	C13—C12—C11	118.10 (12)
C13—N4—C9	117.30 (11)	C13—C12—H12	121.0
Cg1—C1—N2	56.48 (7)	C11—C12—H12	121.0
Cg1—C1—N1	54.29 (7)	N4—C13—C12	124.11 (12)
N2—C1—N1	110.76 (10)	N4—C13—H13	117.9
Cg1—C1—C14	177.65 (13)	C12—C13—H13	117.9
N2—C1—C14	123.84 (11)	C15—C14—C19	119.16 (11)
N1—C1—C14	125.36 (11)	C15—C14—Cg3	59.71 (7)
Cg1—C2—N2	54.52 (6)	C19—C14—Cg3	59.46 (7)
Cg1—C2—C3	55.36 (7)	C15—C14—C1	122.36 (12)
N2—C2—C3	109.88 (10)	C19—C14—C1	118.47 (11)
Cg1—C2—C9	170.96 (12)	Cg3—C14—C1	177.47 (11)
N2—C2—C9	116.46 (11)	C16—C15—C14	119.99 (13)
C3—C2—C9	133.65 (11)	C16—C15—H15	120.0
Cg1—C3—N1	52.60 (7)	C14—C15—H15	120.0
Cg1—C3—C2	51.93 (7)	C17—C16—C15	120.57 (12)
N1—C3—C2	104.53 (10)	C17—C16—H16	119.7
Cg1—C3—C4	169.78 (11)	C15—C16—H16	119.7
N1—C3—C4	117.70 (11)	C16—C17—C18	119.74 (12)
C2—C3—C4	137.66 (11)	C16—C17—H17	120.1
N3—C4—Cg2	61.88 (7)	C18—C17—H17	120.1
N3—C4—C5	121.89 (12)	C19—C18—C17	120.04 (13)
Cg2—C4—C5	60.02 (8)	C19—C18—H18	120.0
N3—C4—C3	114.09 (11)	C17—C18—H18	120.0
Cg2—C4—C3	175.49 (11)	C18—C19—C14	120.48 (12)
C5—C4—C3	123.96 (12)	C18—C19—H19	119.8
C6—C5—C4	118.99 (13)	C14—C19—H19	119.8
C6—C5—H5	120.5	C1—Cg1—C2	142.73 (9)
C4—C5—H5	120.5	C1—Cg1—N1	72.65 (8)
C7—C6—C5	119.64 (13)	C2—Cg1—N1	144.62 (8)
C7—C6—H6	120.2	C1—Cg1—N2	69.79 (8)
C5—C6—H6	120.2	C2—Cg1—N2	72.94 (8)
C6—C7—C8	117.86 (13)	N1—Cg1—N2	142.43 (7)
C6—C7—H7	121.1	C1—Cg1—C3	144.55 (9)
C8—C7—H7	121.1	C2—Cg1—C3	72.71 (8)
N3—C8—C7	124.11 (14)	N1—Cg1—C3	71.92 (8)
N3—C8—H8	117.9	N2—Cg1—C3	145.65 (7)
C7—C8—H8	117.9		
			
C2—N2—C1—Cg1	−0.20 (8)	Cg1—C1—C14—C15	−91 (3)
Cg1—N2—C1—N1	−0.66 (8)	N2—C1—C14—C15	171.95 (12)
C2—N2—C1—N1	−0.87 (14)	N1—C1—C14—C15	−10.5 (2)
Cg1—N2—C1—C14	177.20 (15)	Cg1—C1—C14—C19	90 (3)
C2—N2—C1—C14	176.99 (11)	N2—C1—C14—C19	−7.35 (19)
C3—N1—C1—Cg1	0.83 (8)	N1—C1—C14—C19	170.20 (12)
Cg1—N1—C1—N2	0.68 (8)	Cg1—C1—C14—Cg3	124 (3)
C3—N1—C1—N2	1.51 (14)	N2—C1—C14—Cg3	27 (3)
Cg1—N1—C1—C14	−177.14 (15)	N1—C1—C14—Cg3	−155 (3)
C3—N1—C1—C14	−176.31 (12)	C19—C14—C15—C16	1.3 (2)
C1—N2—C2—Cg1	0.21 (8)	Cg3—C14—C15—C16	0.35 (11)
Cg1—N2—C2—C3	−0.30 (8)	C1—C14—C15—C16	−177.96 (12)
C1—N2—C2—C3	−0.09 (14)	C14—C15—C16—C17	0.1 (2)
Cg1—N2—C2—C9	179.24 (13)	C15—C16—C17—C18	−0.9 (2)
C1—N2—C2—C9	179.44 (11)	C16—C17—C18—C19	0.4 (2)
C1—N1—C3—Cg1	−0.81 (8)	C17—C18—C19—C14	1.0 (2)
Cg1—N1—C3—C2	−0.66 (7)	C15—C14—C19—C18	−1.9 (2)
C1—N1—C3—C2	−1.47 (13)	Cg3—C14—C19—C18	−0.90 (11)
Cg1—N1—C3—C4	176.16 (13)	C1—C14—C19—C18	177.43 (12)
C1—N1—C3—C4	175.36 (11)	N2—C1—Cg1—C2	0.39 (16)
N2—C2—C3—Cg1	0.30 (8)	N1—C1—Cg1—C2	179.63 (13)
C9—C2—C3—Cg1	−179.13 (17)	C14—C1—Cg1—C2	−98 (3)
Cg1—C2—C3—N1	0.67 (8)	N2—C1—Cg1—N1	−179.24 (9)
N2—C2—C3—N1	0.97 (14)	C14—C1—Cg1—N1	82 (3)
C9—C2—C3—N1	−178.46 (13)	N1—C1—Cg1—N2	179.24 (9)
Cg1—C2—C3—C4	−175.16 (18)	C14—C1—Cg1—N2	−98 (3)
N2—C2—C3—C4	−174.86 (14)	N2—C1—Cg1—C3	179.19 (13)
C9—C2—C3—C4	5.7 (3)	N1—C1—Cg1—C3	−1.57 (16)
C8—N3—C4—Cg2	−0.24 (11)	C14—C1—Cg1—C3	81 (3)
C8—N3—C4—C5	−0.53 (19)	N2—C2—Cg1—C1	−0.38 (15)
C8—N3—C4—C3	−177.97 (12)	C3—C2—Cg1—C1	179.27 (14)
Cg1—C3—C4—N3	27.1 (7)	C9—C2—Cg1—C1	−4.7 (8)
N1—C3—C4—N3	9.70 (16)	N2—C2—Cg1—N1	179.00 (13)
C2—C3—C4—N3	−174.85 (14)	C3—C2—Cg1—N1	−1.34 (15)
Cg1—C3—C4—Cg2	1(2)	C9—C2—Cg1—N1	174.7 (7)
N1—C3—C4—Cg2	−16.6 (15)	C3—C2—Cg1—N2	179.65 (9)
C2—C3—C4—Cg2	158.8 (13)	C9—C2—Cg1—N2	−4.4 (7)
Cg1—C3—C4—C5	−150.2 (6)	N2—C2—Cg1—C3	−179.65 (9)
N1—C3—C4—C5	−167.68 (12)	C9—C2—Cg1—C3	176.0 (8)
C2—C3—C4—C5	7.8 (2)	C3—N1—Cg1—C1	−179.04 (10)
N3—C4—C5—C6	0.3 (2)	C1—N1—Cg1—C2	−179.61 (14)
Cg2—C4—C5—C6	0.05 (11)	C3—N1—Cg1—C2	1.35 (15)
C3—C4—C5—C6	177.53 (12)	C1—N1—Cg1—N2	−1.17 (14)
C4—C5—C6—C7	0.2 (2)	C3—N1—Cg1—N2	179.79 (12)
C5—C6—C7—C8	−0.5 (2)	C1—N1—Cg1—C3	179.04 (10)
C4—N3—C8—C7	0.2 (2)	C2—N2—Cg1—C1	179.75 (10)
C6—C7—C8—N3	0.3 (2)	C1—N2—Cg1—C2	−179.75 (10)
C13—N4—C9—C10	0.89 (19)	C1—N2—Cg1—N1	1.19 (14)
C13—N4—C9—C2	179.00 (11)	C2—N2—Cg1—N1	−179.05 (12)
Cg1—C2—C9—N4	−154.6 (7)	C1—N2—Cg1—C3	−179.17 (14)
N2—C2—C9—N4	−158.53 (11)	C2—N2—Cg1—C3	0.59 (15)
C3—C2—C9—N4	20.9 (2)	N1—C3—Cg1—C1	1.58 (16)
Cg1—C2—C9—C10	23.6 (8)	C2—C3—Cg1—C1	−179.24 (14)
N2—C2—C9—C10	19.64 (17)	C4—C3—Cg1—C1	−17.9 (7)
C3—C2—C9—C10	−160.96 (13)	N1—C3—Cg1—C2	−179.18 (9)
N4—C9—C10—C11	−0.4 (2)	C4—C3—Cg1—C2	161.3 (7)
C2—C9—C10—C11	−178.53 (11)	C2—C3—Cg1—N1	179.18 (9)
C9—C10—C11—C12	0.2 (2)	C4—C3—Cg1—N1	−19.5 (6)
C10—C11—C12—C13	−0.5 (2)	N1—C3—Cg1—N2	−179.77 (13)
C9—N4—C13—C12	−1.2 (2)	C2—C3—Cg1—N2	−0.59 (15)
C11—C12—C13—N4	1.0 (2)	C4—C3—Cg1—N2	160.7 (6)

Symmetry codes: (i) *x*+1, *y*, *z*; (ii) −*x*+2, −*y*, −*z*.

### Hydrogen-bond geometry (Å, °)

**Table:**

Cg1, Cg2 and Cg4 are the centroids of the N1/C1,N2,C2,C3, N2/C4–C8 and N4/C9–C13 rings, respectively.

**Table:**

*D*—H···*A*	*D*—H	H···*A*	*D*···*A*	*D*—H···*A*
C15—H15···N4^iii^	0.95	2.59	3.442 (2)	149
N1—H1···N4^iii^	0.89 (2)	2.73 (2)	3.432 (2)	137 (1)
C13—H13···N3^iv^	0.95	2.72	3.505 (2)	141
C16—H16···Cg1^iii^	0.95	2.88	3.671 (1)	142
C7—H7···Cg4^v^	0.95	2.79	3.708 (1)	163
C5—H5···N4	0.95	2.31	3.068 (2)	137
N1—H1···N3	0.89 (2)	2.37 (2)	2.669 (2)	100 (1)
C11—H11···Cg2^vi^	0.95	2.93	3.772 (1)	148

Symmetry codes: (iii) *x*−1/2, −*y*+1/2, *z*−1/2; (iv) *x*+1/2, −*y*+1/2, *z*+1/2; (v) *x*+1/2, −*y*+1/2, *z*−1/2; (vi) −*x*+5/2, *y*−1/2, −*z*+1/2.
